# ACCOUNTING FOR DIVERSITY IN COGNITIVE STATUS IN THE DESIGN AND EVALUATION OF INCLUSIVE PROSPECTIVE MEMORY SOLUTIONS

**DOI:** 10.1093/geroni/igac059.1004

**Published:** 2022-12-20

**Authors:** Edie Sanders, Walter Boot, Robin Stuart

**Affiliations:** Florida State University, TALLAHASSEE, Florida, United States; Florida State University, Tallahassee, Florida, United States; Florida State University, Tallahassee, Florida, United States

## Abstract

Technology holds tremendous promise for supporting older adults' performance of important everyday activities. However, truly inclusive design of technology-based solutions must account for diversity with respect to cognitive status. This talk will focus on empirical studies conducted under the umbrella of the Enhancing Neurocognitive Health, Abilities, Networks, & Community Engagement (ENHANCE) Center with an emphasis on designing inclusive prospective memory solutions for older adults with cognitive impairments. Initial usability studies will be discussed examining the usability and efficacy of novel technology solutions, including the use of smartwatches and digital assistants, to support prospective memory, the ability to remember and carry out an intention in the future, which is crucial for maintaining health, independence, and social connections.

